# Chemical composition, *in vitro* gas production, and nutrient degradability of carob leaves as a sustainable feed for ruminants in Tunisia and Palestine

**DOI:** 10.3389/fvets.2025.1433814

**Published:** 2025-03-10

**Authors:** Soha Ghzayel, Hajer Ammar, Halimeh Zoabi, Bassem Abou Aziz, Ahmed E. Kholif, Moyòsore J. Adegbeye, Rym Ben Abdallah, Mario de Haro-Martí, Secundino Lopez, Mireille Chahine

**Affiliations:** ^1^Department of Biotechnology, National Agriculture Research Center, Ministry of Agriculture, Jenin, Palestine; ^2^Higher Agriculture School of Le Kef, University of Jendouba, El Kef, Tunisia; ^3^Laboratoire des Substances Naturelles, Institut National de Recherche et d’Analyse Physicochimique (INRAP), Sidi Thabet, Tunisia; ^4^Ecole Supérieure d’Agriculture de Mograne, University of Carthage Tunisia, Zaghouan, Tunisia; ^5^National Research Center, Beit Qad Agricultural Station, Jenin, Palestine; ^6^Department of Epidemiology, Ministry of Agriculture, Jenin, Palestine; ^7^Department of Animal Sciences, North Carolina Agricultural and Technical State University, Greensboro, NC, United States; ^8^Dairy Science Department, National Research Centre, Giza, Egypt; ^9^Department of Animal Production and Health, University of Africa, Toru-Orua, Nigeria; ^10^Ecochimie Laboratory (LR21ES02), Department of Biological and Chemical Engineering, National Institute of Applied Sciences and Technology (INSAT), University of Carthage, Tunis, Tunisia; ^11^Gooding County Extension, University of Idaho, Gooding, ID, United States; ^12^Instituto de Ganadería de Montaña (CSIC-Universidad de León), León, Spain; ^13^Departamento de Producción Animal, Universidad de León, León, Spain; ^14^Department of Animal, Veterinary and Food Sciences, University of Idaho, Twin Falls, ID, United States

**Keywords:** carob, degradability, *in vitro* rumen fermentation, methane, phytogenics

## Abstract

**Introduction:**

Carob leaves may be a potential roughage source for ruminants in arid areas. The nutritive value of this feedstuff may be considerably enhanced by the application of solid-phase chemical treatments. This study aimed to evaluate the nutritive value of carob leaves collected from Tunisia and Palestine untreated or treated with urea or sodium hydroxide (NaOH), or supplemented with polyethylene glycol (PEG) on chemical composition and *in vitro* ruminal fermentation.

**Methods:**

Carob leaf samples were collected from either Palestine or Tunisia, and were used either untreated (control) or treated with urea, NaOH at 4% or PEG at 100 mg/g (dry matter (DM) basis), and analyzed for chemical composition. Carob leaves were incubated *in vitro* in diluted rumen fluid fermentation for 48 h, measuring fermentation gasses [methane (CH_4_), and carbon dioxide (CO_2_)], DM degradability and fermentation kinetics.

**Results and discussion:**

Results showed a significant country × treatment interaction for most measured parameters, indicating that treatment effects are constrained by the origin of the leaves. Palestine untreated carob leaves had higher (*p* < 0.001) crude fat, crude protein (CP) and neutral detergent fiber (NDF), but less nonstructural carbohydrate (NSC), acid detergent fiber (ADF) and acid detergent lignin than Tunisia leaves. Tunisia carob leaves had higher concentration (*p* < 0.01) of flavonoids and tannins than leaves from Palestine. Of the three treatments tested, the addition of PEG increased (*p* < 0.01) the gas production during the incubation in diluted rumen fluid of carob leaves and this effect was greater with leaves from Palestine than with those from Tunisia. The other treatments had less noticeable effects, which were different when applied to the leaves from one or another country, given the significance of the interaction country × treatment detected for most of the variables studied. PEG, NaOH and urea treatments of carob leaves can be applied to enhance the ruminal fermentation and energy value of this feedstuff. However, the effects of these treatments are highly dependent on the parent material, and seem to be more effective when applied to a low digestible material.

## 1 Introduction

The high cost of feed represents a significant constraint on global animal production. Recently, researchers have turned to alternative feed resources as a potential cost-effective source of animal fodder, aiming to reduce the production costs of animal products (1–3). There is an interest in bringing agroforestry into animal nutrition through the use of forestry resources in ruminant feed to gain competitive pricing compared to other products (1, 4). Among the promising plants for animal feed, the carob tree stands out. The carob tree (*Ceratonia siliqua*) is an agro-silvopastoral species of valuable socio-economic and ecological interest (5). It thrives in arid and semi-arid zones due to its adaptability to water constraints (6). The carob tree is gaining interest not only for the hardiness and quality of its wood, but also for most of its botanical parts, mainly for its fruits (pods and seeds) and leaves, which are utilized as animal feed (seeds also as a human food). Other parts of the tree are also exploited, such as the flower for carob honey production and the bark and roots for tanning due to their tannin content (2).

Carob fruits stand out for its high carbohydrate content (including primary soluble sugars like sucrose, fructose or glucose), dietary fiber, and polyphenols. It has lower levels of ash, lipids, and protein. Carob leaves, in particular, are highly appetizing and easily consumed by growing lambs (7). The *in vitro* gas production (GP) procedure is helpful for quickly screening feedstuffs and assessing their potential as energy sources for ruminants (8). Numerous studies have reported positive influences of various carob plant parts, such as seeds, pulps, and pods, on ruminants, yielding favorable results (9). Obeidat et al. (7) highlighted the positive effect of carob on milk yield, while digestibility was influenced by the proportion of carob pods in the diet. However, limited research has focused on carob leaves as potential ruminant feed. Additionally, many studies examining carob plants in ruminants have overlooked comparing the regional influence on carob effects in ruminants. Richane et al. (1) demonstrated that geographical location can significantly influence the chemical composition and bioactive components of feedstuffs. Furthermore, carob is noted for its tannin concentration, underscoring the need to investigate how different treatment strategies can effectively reduce tannin content in leaves before utilizing them to feed ruminants.

Several chemical treatments have been proposed to enhance the fermentation and degradability of agriculture byproducts for better valorization (10). Alkali and urea treatments pose no significant risk to animal health and can be used to improve the nutritional value of low-quality roughage (11, 12). Sodium hydroxide (NaOH) treatment breaks ester bonds between lignin and compounds such as acetic acid (C_2_), phenolic acids, cellulose, and hemicellulose in the cell walls through saponification (12). This treatment also falls apart the plant cell wall making the polysaccharides (e.g., cellulose and hemicellulose) more accessible to hydrolytic enzymes for microbial digestion (11, 12). Urea treatment is another method for improving the nutritive value of low-quality forage. Urea treatment increases the nitrogen content of the forage and causes changes in the structure of the cell wall (13). Polyethylene glycol (PEG), a polyether, has a high ability to form stable complexes with tannins, thereby preventing the binding of tannins to proteins. It is widely used to mitigate the negative effects of condensed tannins in ruminant diets (12, 14). Brown and Ng’ambi (14) observed that supplementing *Acacia karroo* leaf meal with PEG at 23 or 30 g increased feed intake in goats without affecting the apparent digestibility of all nutrients or the final body weights of the goats. Recently, Zoabi et al. (12) demonstrated that NaOH treatment of almond hulls reduced crude protein (CP) and both structural and nonstructural carbohydrates (NSC), while urea treatment decreased the fiber fraction and increased CP content.

Consequently, the objectives of the present experiment were to evaluate the nutritive value of carob leaves from Tunisia and Palestine, both untreated and treated with urea or NaOH, or supplemented with PEG, focusing on *in vitro* GP, methane (CH_4_) production, and *in vitro* ruminal fermentation. Our hypothesis was that the nutritive value of carob leaves would vary depending on the origin (country) and that treatment with NaOH or urea, or supplementation with PEG, would enhance the nutritive value of carob leaves as a ruminant feed.

## 2 Materials and methods

### 2.1 Sampling of carob leaves

Samples of leaves of *Ceratonia siliqua* L. (carob) were collected during September–October 2022 either (i) from a mountain area in Tunisia or (ii) from Jenin in Palestine. In Tunisia, samples were collected in the Parc National Djebel Zaghouan, a protected area of 1881 ha in the mountain of Zaghouan (latitude 36°24′10” N, longitude 10°8′35″ E and a peak altitude of 1,295 m above sea level) and covered mainly by Mediterranean forests and shrub lands. The average annual rainfall is 350 mm corresponding to a semi-arid Mediterranean climate. The soils are calcite characterized by a fragile structure. Sampling was stratified by altitude delimiting three separate areas at 250, 400 or 900 m altitude. In Palestine, samples were collected in the Um al-Tout Nature Reserve, a protected area of 36.3 ha in the southern part of Jenin, West Bank (latitude 32°27′33” N, longitude 35°18′03″ E and a peak altitude of 300 m above sea level). The average annual rainfall is 400–500 mm. Sampling was also stratified by altitude in three separate areas at around 90, 140 or 250 m altitude. At each country, leaves (without petioles) were harvested from four carob trees with scissors within each separate sampling area. Leaves were collected daily over the course of a week. Carob leaves collected from the trees of the same sampling area during the collection period (7 samples) were then mixed, and one composite sample was obtained per area, resulting in three independent samples (replicates, one per sampling area) per country. In the laboratory and after each collection, leaves were air-dried at room temperature (with daily air temperatures ranging between 17 and 32°C during the day, similar in both countries at this season of the year) for 1 week and then stored in a dark room at room temperature. Then, three samples of carob leaves were collected from each country (one from each sample area) and all the samples from both countries were delivered to the National Research Centre (NRC) in Giza (Egypt) for further evaluation.

### 2.2 Treatments of carob leaves

In the NRC (Egypt), all received samples were ground using a blender mill (Grindomix GM 300, Normandie-Labo, Normandy, France) with a 1 mm sieve to achieve a uniform particle size. Air-dried carob leaves from Tunisia and Palestine were then treated with either urea or NaOH, while a portion remained untreated as a control. A 4% solution of urea or NaOH in water was applied at a rate of 1 L per kg of dried carob leaves. The treated material was packed into polythene bag silos and manually compressed to expel as much air as possible, minimizing the risk of fungal contamination. The treated biomass was stored in polyethylene bags for 40 days at room temperature (approximately 27°C). After the storage period, samples were oven-dried at 55°C for 48 h, ground, and stored in plastic bags for further analysis and *in vitro* fermentation. Proximate composition analysis and *in vitro* rumen fermentation were conducted at NRC (Giza, Egypt).

### 2.3 *In vitro* fermentation and biodegradation

The *in vitro* fermentation medium was prepared following the method of Goering and Van Soest (15). The detailed procedures for ruminal collection, the in vitro fermentation process, and gas collection for CH_4_ and carbon dioxide (CO_2_) production were previously described by Zoabi et al. (12), and Morsy et al. (16). Briefly, untreated or treated (with either urea or NaOH) leaves (three replicates per country and treatment) were evaluated in two incubation runs, with three bottles per replicate in each run. The untreated leaves were incubated either alone (control) or with the addition of PEG to the incubation medium at a rate of 100 mg PEG/g feed dry matter (DM). In each incubation run 2 blank bottles containing inoculum but no feed were included to establish baseline fermentation GP.

A sample of 1 g ± 10 mg carob leaves (untreated or treated with NaOH or urea) was weighed into filter bags (ANKOM F57; Ankom Technology, Macedon, NY, USA) and placed into 250 mL ANKOM bottles (Ankom^RF^ Gas Production System) equipped with an automatic wireless *in vitro* GP module (Ankom Technology, Macedon, NY, USA) with pressure sensors. Gas pressure was recorded every 10 min for 48 h, and the volume of gas produced by fermentation was calculated. After 48 h of incubation, CH_4_ and CO_2_ concentrations were measured in the gas contained in the bottle headspace using a Gas-Pro detector (Gas Analyzer CROWCON Model Tetra3, Abingdon, UK).

### 2.4 Sampling at the end of incubation and analysis of fermentation end-products

After 48 h of incubation, a sample of the liquid in the bottle was collected to measure pH, analyze total and individual short-chain fatty acid (SCFA) concentrations, and then all the contents were collected to determine the undigested residue to calculate degradability following the procedures previously described by Kholif et al. (17). Total gas and CH_4_ production were expressed relative to degraded DM, neutral detergent fiber (NDF), and acid detergent fiber (ADF) after 48 h of incubation.

### 2.5 Chemical analysis

Samples of carob leaves (treated or untreated) were analyzed for the concentrations of proximate composition and of secondary metabolites as previously detailed by Kholif et al. (18). Methods described by AOAC (19) and Van Soest et al. (20) were used to determine the chemical composition. Plant extracts were obtained from finely ground dry carob leaves and used to determine plant secondary metabolites at the Ecochimie Laboratory, Department of Biological and Chemical Engineering, National Institute of Applied Sciences and Technology (INSAT), University of Carthage, Tunisia. The secondary metabolites analyzed were: total polyphenolics (Folin–Ciocalteu colorimetric method), antioxidant activity using 2,2-diphenyl-1-picrylhydrazyl (DPPH) as a free radical, condensed tannins [modified vanillin assay of Sun et al. (21)], total flavonoids [method of Dewanto et al. (22)], and anthocyanin [method of Gould et al. (23)].

### 2.6 Calculations and statistical analyses

The calculations of GP kinetic parameters including the asymptotic GP (*A*; mL/g DM); the fractional rate of GP (*c*; /h), and the discrete lag time (*Lag*; h) as well as the partitioning factor after 48 h of incubation (PF_48_; mg degradable DM per mL gas), the metabolizable energy (ME) and microbial CP (MCP) production were previously described by Kholif et al. (24).

Data of chemical composition and fermentation were analyzed using the GLM procedure of SAS in a factorial experimental design with the model: *Y_ijk_* = *μ* + *T_i_* + *R_j_* + *(T × R)_ij_* + *ε_ijk_* where: *Y_ijk_* is each individual observation, μ is the population mean, *T_i_* is the treatment effect, *R_j_* is the effect of country (Palestine or Tunisia), *(T × R)_ij_* is the effect of treatment and country interaction, and *ε_ijk_* is the residual error. The treatment effect had three levels (untreated, urea or NaOH) for chemical composition and four levels (untreated, urea, NaOH, PEG) for *in vitro* rumen fermentation studies (PEG was not used as a treatment for the leaves, but added to the medium when untreated leaves were incubated). The experimental unit was the composite sample of carob leaves collected from each sampling area in each country, resulting in three replicates per country.

## 3 Results

### 3.1 Chemical composition and secondary metabolites

Tables 1, 2 show chemical composition and secondary metabolite concentrations of carob leaves under different treatments. There was a significant (*p* < 0.01) country × treatment interaction for all chemical fractions (Table 1). The untreated carob leaves from Palestine had significantly higher (*p* < 0.001) contents of EE, CP, and NDF compared to untreated carob leaves from Tunisia. Specifically, CP and EE in Palestinian untreated carob leaves were 1.89- and 2.79-fold higher than those in Tunisian untreated leaves, respectively. Conversely, untreated leaves from Tunisia showed higher (*p* < 0.001) OM, NSC, ADF, and acid detergent lignin (ADL) than Palestinian untreated leaves.

**Table 1:** Chemical composition of carob leaves untreated (Control) or treated with sodium hydroxide (NaOH), or urea (g/kg DM, unless otherwise stated).

Country	Palestine			Tunisia			SEM	*p* value		
Treatment	Control	NaOH	Urea	Control	NaOH	Urea		Country	Treatment	Country × Treatment
DM	930.2^a^	927.8^a^	931.1^a^	827.0^c^	902.9^ab^	877.0^b^	9.45	<0.001	0.006	0.005
OM	939.7^b^	886.9^e^	936.8^c^	966.1^a^	892.7^d^	965.5^a^	0.81	<0.001	<0.001	<0.001
EE	43.8^c^	50.3^a^	48.6^b^	15.7^d^	10.5^f^	12.9^e^	0.46	<0.001	0.125	<0.001
CP	98.4^b^	77.2^c^	171.7^a^	52.2^d^	45.8^e^	102.6^b^	1.67	<0.001	<0.001	<0.001
NSC	313.8^f^	363.4^d^	237.5^e^	552.0^a^	477.4^c^	505.8^b^	7.48	<0.001	<0.001	<0.001
NDF	483.8^a^	396.0^b^	479.1^a^	346.2^c^	359.0^c^	344.1^c^	4.95	<0.001	<0.001	<0.001
ADF	226.1^e^	252.9^b^	242.4^cd^	237.4^d^	282.5^a^	248.7^bc^	2.87	<0.001	<0.001	0.004
ADL	125.7^e^	153.2^c^	143.4^d^	166.3^b^	174.2^a^	163.6^b^	1.99	<0.001	<0.001	0.003

Means in the same row with uncommon letters are significantly (*p* < 0.05) different. ADF is acid detergent fiber; CP is crude protein; DM is dry matter; EE is ether extract; NDF is neutral detergent fiber; NSC is nonstructural carbohydrates; OM is organic matter; NSC is nonstructural carbohydrates; SEM is standard error of the mean. Multiple mean comparisons were presented only when the interaction was significant.

**Table 2:** Secondary metabolites of carob leaves.

Country	Polyphenol	Anthocyanin	Flavonoid	Tannins	Antioxidant activity^1^		IC_50_ _DPPH
					TEAC, Mmol/L	TEAC, μmol/g	
Palestine	9.91	0.734	17.3	6,740	0.426	1.28	82.7
Tunisia	9.21	0.730	18.3	16,825	0.469	1.41	84.3
SEM	0.467	0.0150	0.12	88.6	0.0071	0.021	0.28
*p* value	0.348	0.863	0.004	<0.001	0.013	0.013	0.013

SEM, Standard error of the mean. IC_50_ _DPPH (mg/mL): the inhibitory concentration of the sample needed to inhibit 50% of the DPPH radicals.

Polyphenol contents in mg/g equivalent gallic acid (GAE)/g DM.

Anthocyanin contents: μg/mL.

Flavonoid contents in catechin equivalent per 100 g DM (CE/100 g).

Tannins contents: catechin equivalent/100 g (CE/100 g DM).

1 Expressed as trolox equivalent antioxidant capacity (TEAC) in both Mmol/L and μmol/g.

The CP content was lowest and ADL highest with NaOH treatment, and this trend was similar in leaves collected from both countries. In contrast, the treatment with urea increased by almost 2-fold the CP content of carob leaves from both countries. As for NDF, its content was reduced in leaves from Palestine treated with NaOH, but this effect was not observed with leaves from Tunisia, in which NaOH or urea treatment had no significant (*p* > 0.05) effects on NDF compared with the control. NaOH-treated leaves exhibit the highest ADF and ADL contents in leaves from both countries. For Tunisian carob leaves, untreated leaves had the highest (*p* < 0.001) values for EE, and NaOH-treated leaves the highest (*p* < 0.006) ADF and ADL.

Table 2 shows the secondary metabolite concentrations in carob leaves collected from both countries. No significant differences (*p* > 0.05) were observed in polyphenol and anthocyanin contents between carob leaves from Palestine and Tunisia. However, there were significant differences (*p* < 0.005) in flavonoids and tannins, with Tunisian carob leaves having higher values. Tannin content in Tunisian carob leaves was 2.5-fold higher (*p* < 0.001) than that in Palestinian leaves. Similarly, antioxidant activity [trolox equivalent antioxidant capacity (TEAC, Mmol/L and μmol/g)] and IC50 _DPPH (mg/mL) in carob leaves from Tunisia were higher (*p* = 0.013) than in those from Palestine.

### 3.2 Total fermentation gas, methane and carbon dioxide production

**Figure 1:**
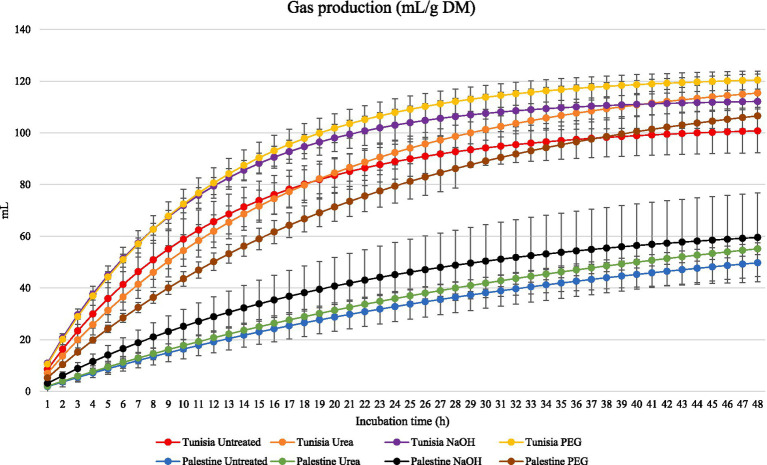
*In vitro* ruminal gas production (mL/g incubated DM) of carob leaves from Palestine or Tunisia untreated or treated with polyethylene glycol (PEG), sodium hydroxide (NaOH), or urea for 48 h of incubation. Error bars represent the variability between the three samples collected form each country and among incubation runs.

Figure 1 illustrates the *in vitro* ruminal GP (mL/g incubated DM) of carob leaves from Palestine or Tunisia untreated (Control) or treated with NaOH, or urea or supplemented with PEG for 48 h of incubation. The overall trend indicates a continuous increase in GP throughout the incubation time. The untreated, and urea and NaOH treated Palestine leaves produced less fermentation gas than Tunisian leaves at any incubation time. When carob leaves from Palestine were incubated with PEG, GP was increased compared with the untreated leaves. With leaves collected in Tunisia, the addition of PEG also increased GP compared with untreated leaves, but this effect was limited and non-significant.

Table 3 details the *in vitro* rumen GP kinetics of carob leaves from Palestine or Tunisia, untreated (control) or treated with PEG, NaOH, or urea. There were significant (*p* < 0.05) interactions between country and treatment for the asymptotic GP and total GP at 48 h of incubation. Gas production was higher when leaves from Tunisia were incubated in comparison with leaves from Palestine except for PEG treated leaves for which GP was similar regardless the origin. With leaves from Tunisia there were no treatment differences in *A* parameter, whereas total GP was increased with PEG addition compared with untreated leaves. With leaves from Palestine, both *A* parameter and total GP were increased with PEG compared with the other treatments. The fermentation rate (parameter *c*) was higher with NaOH than in untreated Palestinian leaves, whereas for Tunisian leaves the only significant difference was that rate was slower in urea-treated than in untreated leaves. The country by treatment interaction did not significantly (*p* = 0.243) affect the lag time.

**Table 3:** *In vitro* rumen gas production (GP) kinetics^1^ of carob leaves from Palestine or Tunisia untreated (Control) or treated with polyethylene glycol (PEG), sodium hydroxide (NaOH), or urea after 48 h of incubation^2^.

Country	Palestine				Tunisia				SEM	*p* value		
Treatment	Control	NaOH	PEG	Urea	Control	NaOH	PEG	Urea		Country	Treatment	Country × Treatment
*A*	70.0^b^	66.5^b^	121.0^a^	78.1^b^	103.0^a^	113.1^a^	122.1^a^	124.0^a^	7.26	<0.001	0.008	0.021
*c*	0.027	0.049	0.045	0.027	0.087	0.101	0.090	0.060	0.0065	<0.001	0.009	0.231
Lag	2.52	0.75	0.78	0.91	1.92	1.63	1.72	2.23	0.483	0.081	0.169	0.243
Total GP	49.7^c^	59.6^c^	106.6^ab^	55.2^c^	100.8^b^	112.2^ab^	120.4^a^	115.4^ab^	5.35	<0.001	<0.001	0.002

Means in the same row with uncommon superscripts are significantly (*p* < 0.05) different. *p*-value is the observed significance level of the *F*-test for treatment; SEM, standard error of the mean. Multiple mean comparisons were presented only when the interaction was significant.

1 GP parameters: *A* is the asymptotic GP (mL/g DM), *c* is the rate of GP (/h), Lag is the initial delay before GP begin (h).

2 Carob leaves untreated (Control treatment) or treated with sodium hydroxide (NaOH treatment) or urea (Urea treatment) or supplemented with polyethylene glycol (PEG treatment).

Table 4 presents the CH_4_ and CO_2_ fermentation outputs of carob leaves from Palestine or Tunisia, either untreated (Control) or treated with PEG, NaOH, or urea after 48 h of incubation. Significant (*p* < 0.05) interactions between country and treatment were observed for CH_4_ production and the proportions of CH_4_ and CO_2_ in total GP. The results indicate that there were not significant differences among treatments in CH_4_ production (per g degradable substrate) or CH_4_ concentration in total gas when carob leaves from Tunisia were incubated. However, with leaves from Palestine CH_4_ was higher when PEG was added to untreated leaves. Overall, CO_2_ concentration in fermentation gas from Tunisian carob leaves was not significantly affected by treatment (*p* > 0.05), whereas with leaves from Palestine treated with urea CO_2_ percentage in total GP was higher than with the untreated leaves.

**Table 4:** Methane (CH_4_) and carbon dioxide (CO_2_) production^1^ of carob leaves from Palestine or Tunisia untreated (Control) or treated with polyethylene glycol (PEG), sodium hydroxide (NaOH), or urea after 48 h of incubation^2^.

Country	Palestine				Tunisia					*p* value		
Treatment	Control	NaOH	PEG	Urea	Control	NaOH	PEG	Urea	SEM	Country	Treatment	Country × Treatment
CH_4_ mL/g DM	14.7^e^	17.3^de^	39.3^a^	14.9^e^	24.8^cd^	32.9^abc^	27.3^bc^	34.4^ab^	2.94	0.001	0.003	0.003
CH_4_ mL/g degradable DM	27.8^c^	37.1^ab^	66.7^a^	33.4^c^	39.7^ab^	50.5^b^	42.1^ab^	53.9^ab^	5.13	0.163	0.009	0.002
CH_4_ mL/g degradable NDF	29.4^d^	38.4^d^	71.3^a^	34.7^cd^	42.8^bcd^	53.8^abc^	46.2^bcd^	58.5^ab^	5.72	0.109	0.010	0.003
CH_4_ mL/g degradable ADF	33.2^c^	41.3^bc^	88.2^a^	32.9^c^	45.8^bc^	58.4^b^	50.4^bc^	63.2^b^	6.68	0.256	0.003	0.006
CH_4_% of total GP	29.3^ab^	28.7^b^	36.7^a^	26.7^b^	24.7^b^	29.3^ab^	22.7^b^	30.0^ab^	2.39	0.046	0.719	0.011
CO_2_ mL/g DM	31.2	40.9	64.5	38.3	73.7	77.3	90.2	78.5	4.06	<0.001	<0.001	0.209
CO_2_ mL/g degradable DM	58.2	88.4	109.8	85.5	117.8	120.2	139.1	123.0	8.43	<0.001	0.005	0.297
CO_2_ mL/g degradable NDF	61.6	91.3	116.8	88.8	126.7	127.7	152.9	134.7	10.16	<0.001	0.009	0.466
CO_2_ mL/g degradable ADF	69.1	97.5	144.7	84.4	135.7	138.8	166.9	144.7	10.58	<0.001	0.001	0.189
CO_2_% of total GP	62.5^bc^	68.9^ab^	60.7^c^	69.8^a^	73.1^a^	68.9^ab^	74.9^a^	67.9^ab^	2.17	0.002	0.919	0.004

Means in the same row with uncommon letters are significantly (*p* < 0.05) different. *p*-value is the observed significance level of the *F*-test for treatment; SEM, standard error of the mean. Multiple mean comparisons were presented only when the interaction was significant.

1 DM is dry matter, NDF is neutral detergent fiber, ADF is acid detergent fiber, GP is gas production.

2 Carob leaves untreated (Control treatment) or treated with sodium hydroxide (NaOH treatment) or urea (Urea treatment) or supplemented with polyethylene glycol (PEG treatment).

### 3.3 Degradability and fermentation

Table 5 provides insight into the *in vitro* rumen fermentation profile and degradability of carob leaves. Significant (*p* < 0.05) country × treatment interactions were observed for DM degradation, C_2_, C_3_, C_2_/C_3_ ratio, pH, ME, and PF_48_. DM degradability was lower in NaOH or urea treated- than in untreated leaves from Palestine, but no significant differences among treatments were observed with leaves from Tunisia. Fiber degradability was not significantly (*p* > 0.05) influenced by any treatment. SCFA increased with PEG addition when leaves from both countries were incubated. With leaves from Tunisia, urea treatment increased C_2_ and C_2_/C_3_ ratio compared with the untreated leaves. In contrast, with leaves from Palestine, treatment of leaves with NaOH or the addition of PEG resulted in lower C_3_ and in higher C_2_ and C_2_/C_3_ ratio compared with the untreated or urea-treated leaves. Fermentation parameters showed that within each country untreated leaves showed the highest pH and the lowest ME. In Palestine-sourced leaves, PF_48_ was greatest for the untreated carob leaves, whereas no differences were observed among treatments in the Tunisian leaves.

**Table 5:** *In vitro* rumen fermentation profile and degradability of carob leaves from Palestine or Tunisia untreated (Control) or treated with polyethylene glycol (PEG), sodium hydroxide (NaOH), or urea after 48 h of incubation^1^.

Country	Palestine				Tunisia				SEM	*p* value		
Treatment	Control	NaOH	PEG	Urea	Control	NaOH	PEG	Urea	SEM	Country	Treatment	Country × Treatment
Degradability^2^												
DM	0.548^b^	0.457^c^	0.587^ab^	0.457^c^	0.625^ab^	0.648^a^	0.648^a^	0.639^a^	0.0251	<0.001	0.046	0.034
NDF	0.515	0.445	0.552	0.443	0.582	0.610	0.593	0.587	0.0254	<0.001	0.157	0.078
ADF	0.470	0.414	0.446	0.462	0.545	0.561	0.542	0.545	0.0267	<0.001	0.876	0.549
SCFA^3^												
Total	23.7	24.0	24.7	23.8	25.0	25.3	25.7	25.0	0.12	<0.001	<0.001	0.403
C_2_	10.3^e^	12.3^bc^	12.1^bcd^	10.6^e^	11.7^cd^	11.2^de^	12.7^b^	13.6^a^	0.29	0.002	0.001	<0.001
C_3_	9.71^a^	7.99^bc^	7.69^c^	9.25^abc^	9.49^ab^	9.13^abc^	9.68^a^	8.45^abc^	0.483	0.144	0.175	0.042
C_4_	3.66	3.65	4.99	3.93	3.73	4.93	3.27	2.95	0.549	0.400	0.412	0.072
C_2_/C_3_	1.06^b^	1.57^a^	1.61^a^	1.16^b^	1.24^b^	1.23^b^	1.32^ab^	1.61^a^	0.094	1.000	0.022	0.002
Fermentation^4^												
pH	6.93^a^	6.77^bc^	6.50^d^	6.77^bc^	6.78^b^	6.67^c^	6.76^c^	6.67^bc^	0.034	0.343	<0.001	<0.001
ME	3.65^d^	3.87^cd^	4.92^b^	4.14^c^	4.92^b^	5.26^a^	5.40^a^	5.29^a^	0.107	<0.001	<0.001	0.003
PF_48_	11.19^a^	7.97^bc^	5.53^c^	8.63^b^	6.22^bc^	5.78^c^	5.40^c^	5.54^c^	1.283	<0.001	0.004	0.023
MCP	475.9	358.0	412.6	378.0	429.5	421.4	411.9	435.8	25.83	0.326	0.137	0.150

Means in the same row with uncommon superscripts are significantly (*p* < 0.05) different. *p*-value is the observed significance level of the F-test for treatment; SEM, standard error of the mean. Multiple mean comparisons were presented only when the interaction was significant.

1 Carob leaves untreated (Control treatment) or treated with sodium hydroxide (NaOH treatment) or urea (Urea treatment) or supplemented with polyethylene glycol (PEG treatment).

2 DM is dry matter degradability (g/g incubated), NDF is neutral detergent fiber degradability (g/g incubated), ADF is acid detergent fiber degradability (g/g incubated).

3 SCFA is short chain fatty acids (mmol/g DM), C_2_ is acetate (mmol/g DM), C_3_ is propionate (mmol/g DM), C_4_ is butyrate (mmol/g DM).

4 ME is metabolizable energy (MJ/kg DM), PF_48_ is the partitioning factor at 48 h of incubation (mg degradable DM/mL gas), MCP is microbial CP production (mg/g DM).

## 4 Discussion

### 4.1 Chemical composition

The significant country × treatment interaction for nutrient concentrations indicates that the effect of the treatment was not the same depending on the origin of the leaves, suggesting that regional factors such as climate, soil quality, forage composition, and management practices influence chemical composition and how this is affected by the chemical treatments. This significant interaction implies that the treatment does not have a uniform effect in leaves from different origins, requiring tailored recommendations. The statistical significance confirms that this variation is unlikely due to chance, highlighting the importance of considering regional differences when evaluating treatment effects on nutrient concentrations. As expected, the significant interaction of origin × treatment on chemical composition are also reflected on subsequent significant effects of this interaction on *in vitro* degradability and rumen fermentation. The differences between both countries (Palestine and Tunisia) in the chemical composition of untreated carob leaves can be attributed to various factors, including environmental parameters such as temperature, altitude, and rainfall, as well as considerations related to variety, cultivation practices, harvesting methods, storage conditions, and processing techniques (12). The results align closely with a study on carob pulp conducted by Richane et al. (1).

Notably, the CP contents observed in this study were considerably higher than those reported in a previous research, especially from Tunisia (1, 25). The elevated CP content in the urea-treated carob leaves can be attributed to the nitrogen supplied by the urea (12). Given that the CP of a feed substance is determined by its nitrogen (including the non-protein N), the increased nitrogen content must be due to the urea treatment. The variation in CP between carob leaves from Palestine and Tunisia may stem from genetic variety/cultivar or environmental multiple factors (12), such as genetic diversity, soil nutrient levels, water availability, etc. Another factor contributing to nutrient and plant metabolite variation could be the cultivars, as El Hajaji et al. (26) demonstrated variation in antioxidant and phenolic components among three varieties of carob tree leaves from Morocco. The leaf maturity (phenological) stage before harvesting in both countries could also play a role in the observed differences.

The main disparities in secondary metabolite composition are associated with environmental and natural factors, such as region, and variety (27, 28). Factors like the maturity stage, genetic diversities, or the cultivation environment could contribute to changes in the concentration of plant secondary metabolites. Phenolic compounds in carob leaves, known for their antioxidant properties in scavenging free radicals and preventing oxidative damage to cells, have been identified as effective antioxidants in plant foods, including fruits and vegetables (2). Utilizing this by-product as an energy supply for livestock may thus mitigate the risk of diseases related to oxidative stress (29). Polyphenolic compounds, including tannins, are recognized for forming complex linkages with metal ions and macromolecules such as proteins and polysaccharides (30). In the present study, polyphenol content and antioxidant capacity (IC_50_) of carob leaves were consistent with the literature. The low IC_50_ value and the flavonoid content suggest robust antioxidant activity (31). Furthermore, these compounds are known to have beneficial effects on protein metabolism in ruminants, promoting reduced breakdown of dietary proteins in the rumen and enhancing by-pass protein and the uptake of amino acids in the small intestine (32).

### 4.2 Ruminal fermentation and degradability

The *in vitro* GP method is a reliable tool for animal feed assessment, as GP correlates well with MCP synthesis and *in vivo* and *in vitro* digestibility (33, 34). Pastorelli et al. (2) suggest a relationship between rumen fermentation of organic matter and GP, which aligns with the results of the present study. In this regard, it should be noted that greater GP indicates greater fermentative activity and nutrient degradation by rumen microorganisms. The range in chemical composition led to variability in rumen fermentation kinetics and degradability (35). The GP kinetics for carob leaves collected from Palestine or Tunisia revealed a rapid and significant degradation of carob leaves, which may be associated with their richness in carbohydrates easily bioavailable to the ruminal microbiota (36). The untreated carob leaves from Tunisia were fermented at a faster rate releasing more fermentation gas than those from Palestine, what may be attributed to the higher nonstructural carbohydrates (NSC) content that are more rapidly available to the rumen microbes, leading to increased GP from the substrate (37). The higher *in vitro* digestibility coefficients confirm that untreated carob leaves from Tunisia were fermented to a greater extent than those from Palestine, and the increased nutrient digestibility resulted in higher volatile fatty acid (SCFA) concentration in the medium after in vitro incubation. Carbohydrate fractions affect fermentation kinetics differently. Non fiber carbohydrates favor a more intense and faster fermentation, while on the contrary, structural carbohydrates are degraded to a lesser extent and at a slower rate, limiting the access of microorganisms to cell contents, reducing nutrient degradability (8). Gioxari et al. (38) reported that carob fatty acid composition contains proportions of C12: 0 and C14:0 which can have some antimicrobial activity that could have affected the fermentation from Palestine carob leaves. PEG seemed to be the most effective treatment among those tested to enhance ruminal fermentation of carob leaves, as GP increased when PEG was added to untreated leaves collected from both countries. PEG has been applied to animal diet by various methods such as spraying of tannin rich green leaves, treatment of harvested leaves, infusion into the rumen and drenching of the animal (39). Furthermore, PEG added to tannin containing plant samples increased *in vitro* digestibility of DM and CP (40). Priolo et al. (41) reported that carob pulp supplemented with PEG improved lamb performance, even similar to maize-based diet level by eliminating the effects of condensed tannin. The ability of PEG to bind tannin reduces the formation of protein-tannin complexes (42) thus preventing the anti-nutritional effects of tannins. Polyethylene glycol is a polymer containing many oxygen atoms capable of forming hydrogen bonds with hydroxyl groups of tannins (43). The extent of the effect on ruminal fermentation was greater when PEG was added to leaves from Palestine than to those from Tunisia, indicating that PEG was more effective with low degradable substrates. Both CO_2_ and CH_4_ are important enteric gasses from ruminants. They are generally reported to contribute to the greenhouse gasses (GHG) emissions from livestock. Therefore, studies are consciously looking for options to reduce their emission and perhaps shift emissions from CH_4_ to CO_2_ since CO_2_ GHG warming potential is lower than CH_4_. In this study, the CH_4_ production was higher when total GP was higher, with subtle differences in CH_4_ concentration in the fermentation gas. Some treatment effects on CH_4_ to CO_2_ *in vitro* were observed when leaved from Palestine were incubated, whereas there were no significant differences among treatments with the leaves from Tunisia. The only consistent effect was a higher CH4 production when PEG was added to Palestine-sourced carob leaves, confirming that PEG effects may become more noticeable with low degradable feedstuffs.

A decreased PF_48_ reflects a lower conversion of degraded substrate into microbial biomass (44). The addition of PEG to carob leaves from both countries increased the estimated ME content of these feedstuffs, suggesting that PEG may enhance energy utilization. Silanikove et al. (42) reported that PEG supports enhanced ME and CP availability to ruminants. With leaved from both countries, all the treatments applied increased the calculated ME concentration compared with the untreated leaves. The increase in energy value was greater with the carob leaves collected from Palestine, supporting that these treatments are more effective with less digestible roughages.

## 5 Conclusion

The current study revealed that the chemical composition and potential nutritional value of carob leaves were substantially influenced by geographical origin. Carob (*Ceratonia siliqua* L.) is widely used as a source of fiber for small ruminants in the Mediterranean region. Carob leaves have a potentially great nutritional value for ruminants. The results of this study suggest the possibility of using carob leaves in the diets of small ruminants, with the advantage that, being local natural resources, they are better adapted to the climate and agronomic conditions and limit the impact on the environment. PEG, NaOH and urea treatments of carob leaves can be applied to enhance the ruminal fermentation and energy value of this feedstuff. The effects of these treatments are highly dependent on the parent material, and seem to be more effective when applied to a low digestible material. Further research will be required to establish the most appropriate level of inclusion of this feedstuff in ruminants diets according to their production responses and, therefore, their economic impact.

## Data Availability

The original contributions presented in the study are included in the article/supplementary material, further inquiries can be directed to the corresponding author.
